# Metabolic Effects of Antidepressants: Results of a Randomized Study’s Interim Analysis

**DOI:** 10.7759/cureus.42585

**Published:** 2023-07-27

**Authors:** N Simple Santi, Sashi B Biswal, Birendra Narayan Naik, Jyoti Prakash Sahoo, Bhabagrahi Rath

**Affiliations:** 1 Pharmacology, Veer Surendra Sai Institute of Medical Sciences and Research, Sambalpur, IND; 2 Psychiatry, Veer Surendra Sai Institute of Medical Sciences and Research, Sambalpur, IND; 3 Pharmacology, Kalinga Institute of Medical Sciences, Bhubaneswar, IND

**Keywords:** vortioxetine, vilazodone, serotonin dysfunction, body mass index (bmi ), egfr creatinine, serum triglycerides, total cholesterol levels, fasting blood glucose (fbg), hamilton depression rating scale, depressive disorder

## Abstract

Background and objectives: Individuals with major depressive disorder exhibit a dysregulated metabolic profile. There are few studies on how vilazodone, escitalopram, and vortioxetine alter metabolic parameters. Our study aimed to determine the change in plasma glucose, HbA_1c_, serum cholesterol, triglyceride, and creatinine at 12 weeks.

Methods: An ongoing randomized, open-label, three-arm study's interim analysis is portrayed here. The participants were assessed at baseline, 4, 8, and 12 weeks after receiving oral tablets of either vilazodone (20-40mg/d), escitalopram (10-20mg/d), or vortioxetine (5-20mg/d). This study is CTRI-registered (2022/07/043808).

Results: Of 71 recruited participants, 49 (69%) completed the 12-week visit. The median Hamilton Depression Rating Scale (HDRS) scores of the participants in vilazodone, escitalopram, and vortioxetine groups were 30.0, 29.5, and 29.0 at baseline (p=0.76) and 19.5, 19.5, and 18.0 (p=0.18) at 12 weeks, respectively. The median fasting blood sugar (FBS) values were 98.5, 105.5, and 98.0 at baseline (p=0.07) and 94.0, 99.5, and 96.0 (p=0.19) at 12 weeks, for vilazodone, escitalopram, and vortioxetine groups, respectively. The post hoc analysis did not yield statistically significant differences regarding any parameters.

Conclusion: According to this interim study, the HDRS scores declined after 12 weeks of therapy. The subjects' metabolic parameters did not significantly change. It is essential to perform further investigation regarding these impacts.

## Introduction

Major depressive disorder (MDD) creates havoc on an individual's physical, psychological, and interpersonal life. Through significant neurohormonal mechanisms, MDD and metabolic disorders can coexist [1]. During the preceding twenty years, the prevalence of MDD has fostered a sharp rise around the globe [2]. According to recent statistics, the annual prevalence of MDD is 15.9% in India [3]. The metabolic parameters like blood glucose, serum creatinine, serum total cholesterol, and triglyceride tend to derange as MDD advances. These parameters are somewhat fixed via antidepressants [4-7].

While there exist several effective antidepressants on the shelves, it is unexplored which drug optimizes these metabolic traits. Plus, less information exists about the magnitude and longevity of these amenities [8]. Based on the literature survey, we planned this study with three antidepressant drugs, namely, escitalopram, a selective serotonin receptor inhibitor (SSRI); vilazodone, an SSRI with partial agonistic action at 5-HT1A receptors; and vortioxetine, a serotonin modulator plus transporter inhibitor [9-11]. This study was backed by the contention that newer antidepressants could aid in improving the metabolic parameters of patients with MDD. Recent studies advocate the betterment of such metabolic parameters in patients on regular antidepressants [12-14].

This study aimed to determine the metabolic parameters in patients with MDD after 12-week antidepressant monotherapy of vilazodone, escitalopram, or vortioxetine. Here we reported the interim results of a more extensive ongoing study focused on changes at week 12 in the fasting blood sugar (FBS), glycosylated or glycated hemoglobin (HbA1c), serum total cholesterol, triglyceride, serum creatinine, body mass index (BMI) along with the Hamilton Depression Rating Scale (HDRS) score [15]. We also analyzed the association between the changes in FBS and HDRS at week 12.

## Materials and methods

This randomized, open-label, three-arm, open-label study seeks to determine the effects of vilazodone, escitalopram, and vortioxetine on the metabolic parameters of individuals with MDD. In July 2022, we started recruiting participants in the Department of Psychiatry, Burla, VIMSAR, India. Before group allocation, the participants or their relatives signed written informed consent for the study. Before the study commenced, the Institutional Ethics Committee, VIMSAR, Burla, Odisha, India, provided us with ethical permission (No. 029-2022/I-S-T/03 dated 17.05.2022). Our study is prospectively registered with the Clinical Trial Registry, India (CTRI/2022/07/043808). The study adhered to the International Council for Harmonization's recommendations for Good Clinical Practice, Declaration of Helsinki, and institutional norms.

Study participants

Individuals 18-65 years of age diagnosed with MDD, with an HDRS score of ≥ 24, were included. This study excluded patients with degenerative neurological illnesses, psychotic symptoms, kidney disease (estimated glomerular filtration rate at baseline < 45ml/min/1.73m2), cardiovascular incidents within the preceding six months, serum alanine transaminase (ALT) or aspartate transaminase (AST) levels > 150% the upper limit of normal, serum triglyceride > 400 mg/dl, and pregnant or nursing women. The participants are free to retract their consent at any moment without elucidation.

Study design and endpoints

In a 1:1:1 ratio, the participants received a random selection to commence intervention with tablets of either vilazodone 20-40mg once daily (group A), escitalopram 10-20mg once daily (group B), or vortioxetine 5-20mg once daily (group C). Permuted block randomization with blocks 12 and 24 was adopted to accomplish this. We stratified randomization according to gender (male or female) and disease condition (treatment naïve or on antidepressants for < 6 months. For the assessment involving metabolic parameters in this interim analysis, we sorted the participants as diabetics and non-diabetics.

The change in FBS from baseline at week 12 was the primary objective for this interim analysis. Secondary objectives were changes in the HDRS score, glycated hemoglobin (HbA1c), serum total cholesterol, triglyceride, serum creatinine, and body mass index (BMI) at week 12. The analyses were concentrated on the per-protocol (PP) population.

Study procedure

Throughout the study span, each participant received one of these antidepressant tablets: vilazodone 20-40mg once daily (group A), escitalopram 10-20mg once daily (group B), or vortioxetine 5-20mg once daily (group C). The lead investigator supplied these medications to the participants free of cost. Conforming the participant's clinical outcome to the drugs given, the psychiatrist tuned their dosages. No cross-over was allowed. At the initial excursion, all participants comprehensively assessed their mental and physical health. We scheduled follow-up appointments after 4, 8, and 12 weeks of the baseline visit. At baseline and after that, every four weeks through week 12, the participant's HDRS scores and the abovementioned metabolic parameters were evaluated. We correlated the changes in FBS and HDRS at week 12 from the respective baseline values. Lower HDRS scores infer tremendous therapeutic success and less depressed symptoms.

Statistical analysis

The sample size was finalized for the primary endpoint of the whole study. Considering a mean difference in the HDRS of 10.0 from baseline and a standard deviation of 2.0, 87 patients (29 in each group) were needed to measure a change in the HDRS with an 80% power at a 0.05 two-sided significance level. Ninety-six participants (32 in each group) were finalized for this study considering a 10% attrition rate into account. After completing the 12-week visit by the first 48 subjects, we performed an interim analysis.

To verify the normality of the data, the Shapiro-Wilk test was implemented. The continuous data were displayed as median with interquartile range (IQR), and the Kruskal-Wallis test was applied to gauge them prior to post hoc analysis by the Bonferroni test. The categorical data were shown as the frequency with proportion, and Pearson's chi-square (ꭓ2) test was adopted for their evaluation. For data computation, we leveraged the R software (version 4.2.3) [16]. Each statistical test was two-tailed, and a p-value of 0.05 or lower was treated as statistically significant.

## Results

**Figure 1:**
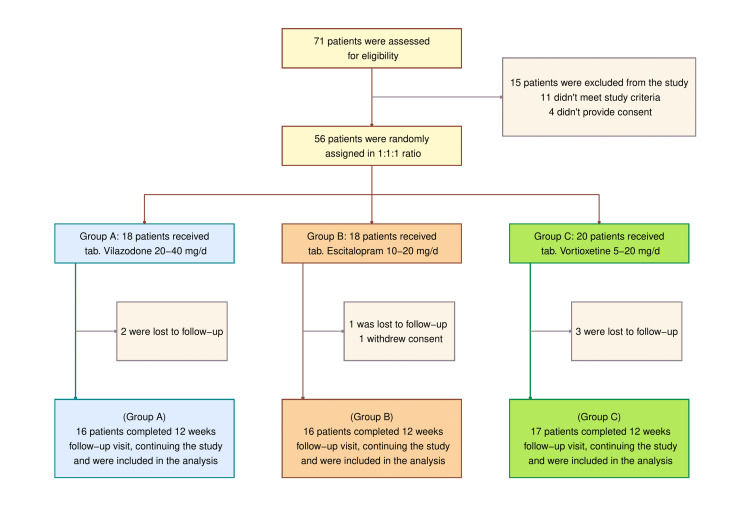
The CONSORT diagram The CONSORT (consolidated standards of reporting trials) diagram shows the screening, randomization, and completion of study visits till the interim analysis. Eleven patients were ineligible according to the study criteria. Ten within 18-65 years of age did not come, and one was 12 weeks pregnant at the baseline visit. This interim analysis was done in the per-protocol (PP) population (n = 49).

For eligibility, 71 patients underwent screening. Four individuals who hesitated to express their consent, one pregnant woman, and 10 others who fell short of the age limits were dropped from the research study. Each of the remaining 56 participants received a random assignment to one of the three study groups. One participant in the escitalopram arm revoked her consent, and six additional individuals (two, one, and three among the pertinent study groups) failed to turn up for their follow-up visits. Forty-nine participants (24 women and 25 men; 16, 16, and 17 in the vilazodone, escitalopram, and vortioxetine arms, respectively) were assessed in this interim analysis (Figure 1). Participants possessed similar baseline traits across the three study groups (Table 1).

**Table 1:** Baseline characteristics of the study population (n = 49) The continuous variables were expressed as the median (interquartile range). The categorical values were presented as n (%). BMI: Body mass index; HDRS: Hamilton Depression Rating Scale-17 items version; FBS: fasting blood sugar; HbA1c: glycated hemoglobin.

Parameters	Total (n = 49)	Group A Vilazodone (n = 16)	Group B Escitalopram (n = 16)	Group C Vortioxetine (n = 17)	p-Value
Age (years)	44.0 (34.0 - 55.0)	47.5 (38.8 - 53.5)	42.0 (33.0 - 54.5)	43.0 (34.0 - 55.0)	0.094
Gender					
Female	25 (51.0%)	8 (50.0%)	8 (50.0%)	9 (52.9%)	0.981
Male	24 (49.0%)	8 (50.0%)	8 (50.0%)	8 (47.1%)	
Presence of diabetes					
Diabetic	23 (46.9%)	6 (37.5%)	10 (62.5%)	7 (41.2%)	0.634
Non-diabetic	26 (53.1%)	10 (62.5%)	6 (37.5%)	10 (58.8%)	
BMI (kg/m^2^)	27.1 (25.6 - 28.5)	26.6 (25.4 - 28.5)	26.9 (26.3 - 27.8)	28.0 (26.0 - 28.6)	0.126
HDRS	30.0 (29.0 - 31.0)	30.0 (29.0 - 31.0)	29.5 (29.0 - 30.0)	29.0 (29.0 - 32.0)	0.763
FBS (mg/dl)	101.0 (93.0 - 122.0)	98.5 (89.5 - 113.3)	105.5 (97.5 - 123.3)	98.0 (93.0 - 124.0)	0.066
HbA_1c_ (%)	6.6 (6.0 - 7.1)	6.4 (5.9 - 7.2)	6.8 (6.5 - 7.1)	6.6 (5.8 - 7.1)	0.287
Serum cholesterol (mg/dl)	154.0 (141.3 - 164.0)	145.0 (130.8 - 158.3)	152.5 (145.8 - 168.3)	159.5 (151.0 - 167.8)	0.102
Serum triglyceride (mg/dl)	120.5 (105.3 - 138.0)	109.5 (102.8 - 126.0)	124.5 (108.3 - 145.0)	127.5 (110.3 - 142.5)	0.316
Serum creatinine (mg/dl)	0.87 (0.80 - 0.94)	0.87 (0.80 - 0.94)	0.86 (0.81 - 0.94)	0.87 (0.73 - 0.93)	0.797

**Figure 2:**
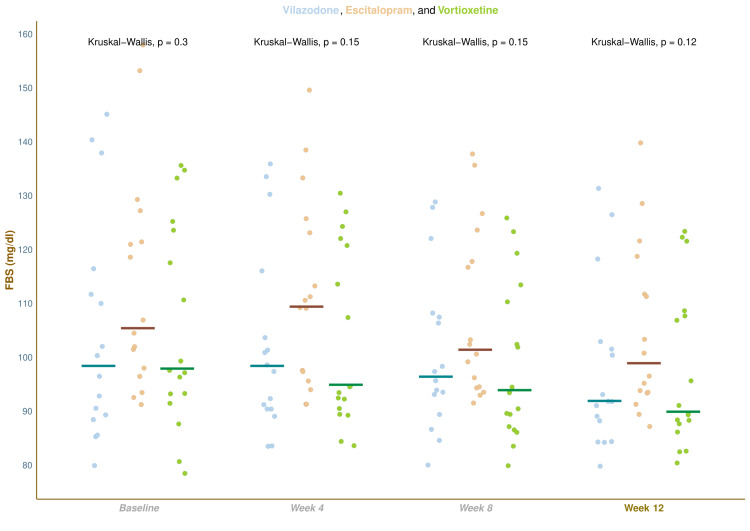
Fasting blood sugar values of the study participants The jitter plots illustrate the fasting blood sugar (FBS) values of all study participants at the baseline as well as the follow-up visits. The horizontal lines represent the corresponding median values. The intergroup comparisons at each visit were made with the Kruskal-Wallis test.

Figure 2 displays the FBS values of the study population. The median FBS values were 98.5 (89.5-113.3) at baseline, 98.5 (90.8-107.0) at four weeks, 96.5 (92.0-107.3) at eight weeks, and 92.0 (87.0-102.3) at 12 weeks in the vilazodone group (mean difference from baseline: -8.5 (95% confidence interval (CI): -14.0 to -0.3); p = 0.016), 105.5 (97.5-123.3) at baseline, 109.5 (96.8-123.8) at four weeks, 101.5 (94.8-119.5) at eight weeks, and 99.0 (93.8-113.8) at 12 weeks in the escitalopram group (mean difference from baseline: -9.5 (95% CI: -13.3 to -4.3); p = 0.021), and 98.0 (93.0-124.0) at baseline, 95.0 (91.0-121.0) at four weeks, 94.0 (87.0-110.0) at eight weeks, and 90.0 (88.0-108.0) at 12 weeks in the vortioxetine group (mean difference from baseline: -9.0 (95% CI: -13.0 to -5.0); p = 0.009), respectively. Except for the transient rise of median FBS value in the escitalopram group at week 4, the blood glucose level of the study participants plummeted gradually. Post hoc analysis with the Bonferroni test revealed that participants in the vortioxetine group had clinically but not statistically significant improvement in the FBS values compared to participants of the other two groups.

**Figure 3:**
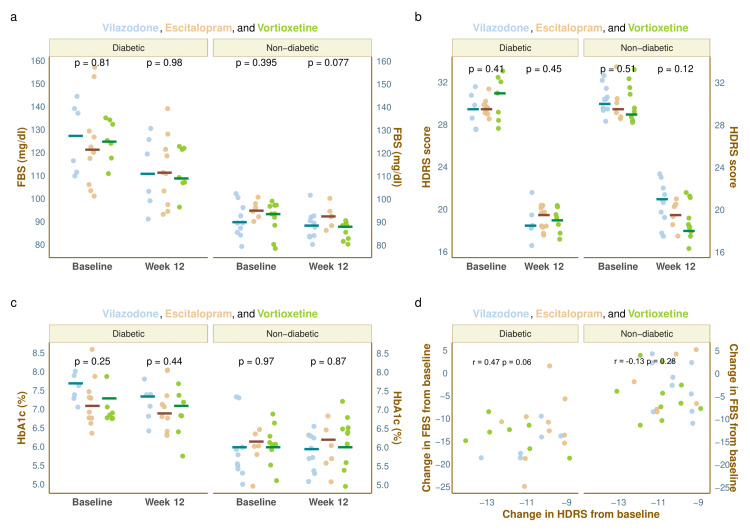
Glycemic parameters and HDRS scores of the study participants The plots illustrate the glycemic parameters and HDRS scores of all study participants (sub-grouped as diabetic or non-diabetic at the baseline visit) at the baseline and week 12. The horizontal lines represent the corresponding median values. The inter-group comparisons at each visit were made with the Kruskal-Wallis test. The jitter plots in parts a, b, and c demonstrate the FBS, HDRS, and HbA1c values of diabetic and non-diabetic participants at the baseline and week 12. The scattered plot in part d depicts the association between the change in FBS values from baseline and the change in HDRS scores among the diabetic and non-diabetic participants. FBS: Fasting blood sugar; HDRS: Hamilton Depression Rating Scale-17 items version; HbA1c: glycosylated hemoglobin.

The indicators of glycemic status, i.e., FBS and HbA1c values and the HDRS scores of the study population at baseline and week 12, are illustrated in Figure 3. Each of the four plots in this figure has been faceted according to the presence or absence of diabetes at the baseline visit. The median baseline FBS values of diabetic individuals in the three study groups were 127.5 (113.3-139.5), 121.5 (109.8-128.5), and 125.0 (121.0-134.0), respectively (p = 0.811). After 12 weeks of intervention, these values were 111.0 (100.8-124.3), 111.5 (98.8-121.3), and 109.0 (107.5-122.0), respectively (p = 0.983). The non-diabetic participants had median baseline FBS values of 90.0 (86.5-96.0), 95.0 (93.3-97.5), and 93.5 (89.0-97.0), respectively (p = 0.395), which got reduced to 88.5 (84.0-91.8), 92.5 (89.8-94.5), and 88.0 (83.8-89.0), respectively (p = 0.077) after 12 weeks of intervention (Figure 3a). The median baseline HDRS scores of diabetic individuals in the three groups were 29.5 (28.3-30.8), 29.5 (29.0-30.0), and 31.0 (29.0-32.0), respectively (p = 0.41). After 12 weeks, the scores were 18.5 (18.0-19.0), 19.5 (18.3-20.0), and 19.0 (18.0-19.5), respectively (p = 0.45). The non-diabetic participants had median baseline HDRS scores of 30.0 (29.3-31.8), 29.5 (29.0-30.0), and 29.0 (28.3-31.0), respectively (p = 0.51), which were decreased to 21.0 (19.3-21.8), 19.5 (19.0-20.8), and 18.0 (18.0-20.5), respectively (p = 0.12) after 12 weeks of intervention (Figure 3b). The median baseline HbA1c values of diabetic individuals in the corresponding groups were 7.7 (7.3-7.8), 7.1 (6.9-7.3), and 7.3 (7.1-7.5), respectively (p = 0.25). After 12 weeks, the values were 7.4 (6.9-7.5), 6.9 (6.7-7.1), and 7.1 (7.0-7.1), respectively (p = 0.44). The non-diabetic participants had median baseline HbA1c values of 6.0 (5.8-6.2), 6.2 (5.8-6.5), and 6.0 (5.8-6.6), respectively (p = 0.97) which were changed to 6.0 (5.8-6.0), 6.2 (5.9-6.5), and 6.0 (5.8-6.5), respectively (p = 0.87) after 12 weeks of intervention (Figure 3c). Post hoc analysis with the Bonferroni test revealed no statistically significant difference among the groups regarding any of the abovementioned parameters. The changes in FBS and HDRS scores at week 12 were positively correlated (r = 0.47, p = 0.06) for diabetic persons and negatively correlated (r = -0.13, p = 0.28) for non-diabetic individuals (Figure 3d).

**Figure 4:**
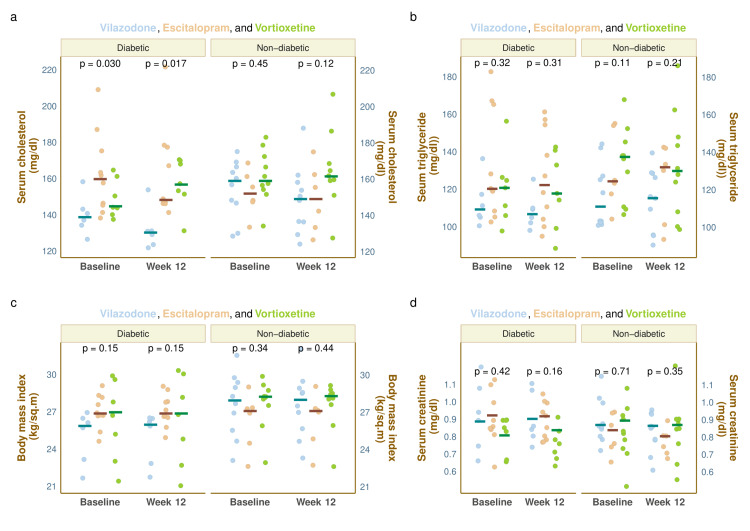
Other metabolic parameters of the study participants The jitter plots illustrate other metabolic parameters of all study participants (sub-grouped as diabetic or non-diabetic at the baseline visit) at the baseline and week 12. The horizontal lines represent the corresponding median values. The intergroup comparisons at each visit were made with the Kruskal-Wallis test. Parts a, b, c, and d demonstrate the serum cholesterol, triglyceride, body mass index (BMI), and serum creatinine values, respectively, of diabetic and non-diabetic participants at the baseline and week 12.

The metabolic parameters, e.g., serum total cholesterol, triglyceride, body mass index, and serum creatinine of the study population at baseline and week 12, are illustrated in Figure 4. Each of the four plots in this figure has been faceted according to the presence or absence of diabetes at the baseline visit. The median baseline serum cholesterol levels of diabetic individuals in the three groups were 139.0 (135.5-142.5), 160.0 (145.3-173.0), and 145.0 (142.5-156.0), respectively (p = 0.030). At 12 weeks, these values were 130.5 (125.5-131.8), 148.5 (147.0-175.5), and 157.0 (152.5-169.0), respectively (p = 0.017). The median baseline serum cholesterol of non-diabetic participants, i.e., 159.0 (147.3-166.0), 152.0 (148.0-160.5), and 159.0 (154.8-170.8), (p = 0.45) was changed to 149.0 (136.0-157.0), 149.0 (136.0-160.5), and 161.5 (159.3-167.0), respectively (p = 0.12) after 12 weeks of intervention (Figure 4a). The median baseline serum triglyceride values of the diabetic participants of the 3 study groups were 109.5 (105.5-115.8), 120.5 (111.5-156.5), and 121.0 (109.0-126.0), respectively (p = 0.32). After 12 weeks of intervention, these values were 107.0 (103.5-109.8), 122.5 (105.8-150.3), and 118.0 (107.5-137.0), respectively (p = 0.31). The median baseline triglyceride values of non-diabetic persons, i.e., 111.0 (102.3-126.8), 124.5 (119.5-146.8), and 137.5 (115.8-144.0), (p = 0.11) were changed to 115.5 (99.3-128.3), 132.0 (109.3-139.8), and 130.0 (112.0-147.0), respectively (p = 0.21) after 12 weeks (Figure 4b). The baseline BMI of the people with diabetes were 25.9 (23.8-26.3), 26.9 (26.7-27.8), and 27.0 (24.0-28.9), respectively (p = 0.15). After 12 weeks, the values were 26.0 (23.7-26.4), 26.9 (26.7-27.7), and 26.9 (24.0-29.0), respectively (p = 0.15). The non-diabetic participants had median baseline BMI of 28.0 (26.0-29.2), 27.1 (25.1-27.3), and 28.3 (27.9-28.6), respectively (p = 0.34), which were changed to 28.0 (26.0-29.0), 27.1 (25.3-27.5), and 28.3 (27.9-28.6), respectively (p = 0.44) after 12 weeks of intervention (Figure 4c). The median baseline serum creatinine values of diabetic individuals in the corresponding groups were 0.89 (0.78-0.97), 0.93 (0.83-0.99), and 0.81 (0.76-0.93), respectively (p = 0.42). After 12 weeks, the values were 0.91 (0.82-0.94), 0.92 (0.87-0.95), and 0.84 (0.72-0.89), respectively (p = 0.16). The non-diabetic participants had a median baseline serum creatinine of 0.87 (0.82-0.94), 0.84 (0.83-0.86), and 0.90 (0.73-0.96), respectively (p = 0.71) which got changed to 0.87 (0.80-0.88), 0.81 (0.79-0.81), and 0.87 (0.76-0.91), respectively (p = 0.35) after 12 weeks of intervention (Figure 4d). Post hoc analysis with the Bonferroni test revealed no statistically significant difference among the groups regarding any of the parameters mentioned earlier.

## Discussion

This interim analysis revealed that all three study medications had analogous effects on the metabolic markers in individuals with diabetes and those without diabetes. Nearly all participants complied with the study protocol and had reduced HDRS scores after 12 weeks of therapy. Differences in HDRS and FBS values had a non-significant correlation. Our findings on metabolic parameters and HDRS scores accorded with those from two previous investigations [8,12].

20-40 mg of vilazodone and 5-20 mg of vortioxetine were the daily doses for the experimental groups, while 10-20 mg of escitalopram was the daily dose for those enrolled in the control arm. Escitalopram, which serves as the control, has only one mechanism of action, while vilazodone has the bonus of being a partial agonist at the 5-HT1A receptor. Vortioxetine, meanwhile, interferes with serotonin receptors and hinders its transport. We noticed that the reduction in HDRS scores and improved quality of life in the vortioxetine group were more significant than in the remaining two groups [17,18]. These results hint that vortioxetine monotherapy may be viable for managing MDD.

Regardless of the groups they were allocated to, all trial participants acquired no-cost medication. The low attrition rate could have ensued from this. All the study participants had plummeted HDRS scores after 12 weeks of antidepressant medications. Nonetheless, neither of the studied drugs prompted any metabolic parameter to change in a way that could be deemed clinically or statistically significant. Per some previous studies, vortioxetine proved its potential to improve the metabolic parameters of patients with MDD [12-14]. However, our results did not align with the studies cited above. According to our preceding analyses, optimal antidepressant activity mandates frequent visits, superior quality of life, and excellent drug compliance [17,18]. This study explores the metabolic parameters and HDRS scores to illuminate what ensued. As mentioned earlier, the findings must be perceived as preliminary, as this was merely an initial audit of a larger research project. Furthermore, additional evaluations are crucial to determine whether the medications have a noticeable impact on the metabolic indicators after an extended follow-up.

Permuted block randomization and subgroup analysis of the study objectives were this study's core strengths. Regular follow-up visits and gauging depression symptoms employing a widely acknowledged and adopted HDRS offered additional advantages [15]. Our study had a few drawbacks, likewise. First, the dropouts and computation issues of the metabolic parameters might be traced to the open-label trial design. Second, the medications utilized for this study were offered without charge. The dearness of the study medications could constrain the practical relevancy of the study findings. Third, the abovementioned findings hinge on an interim analysis of a more extensive and continuing study. It is necessary to explicitly validate the coherence between these results and those from the ongoing larger study.

## Conclusions

According to this interim analysis, after completing the therapeutic intervention for 12 weeks, each participant's HDRS scores significantly decreased. The metabolic indices of the participants were not changed significantly. Further analysis of these effects is imperative.

## References

[1] McIntyre RS, Soczynska JK, Konarski JZ, Kennedy SH (2006). The effect of antidepressants on glucose homeostasis and insulin sensitivity: synthesis and mechanisms. Expert Opin Drug Saf.

[2] Herrman H, Kieling C, McGorry P, Horton R, Sargent J, Patel V (2019). Reducing the global burden of depression: a Lancet-World Psychiatric Association Commission. The Lancet.

[3] Mishra A, Galhotra A (2018). Mental Healthcare Act 2017: need to wait and watch. Int J Appl Basic Med Res.

[4] McIntyre RS, Park KY, Law CW (2010). The association between conventional antidepressants and the metabolic syndrome: a review of the evidence and clinical implications. CNS Drugs.

[5] Deuschle M (2013). Effects of antidepressants on glucose metabolism and diabetes mellitus type 2 in adults. Curr Opin Psychiatry.

[6] Kopf D, Westphal S, Luley CW (2004). Lipid metabolism and insulin resistance in depressed patients: significance of weight, hypercortisolism, and antidepressant treatment. J Clin Psychopharmacol.

[7] Iwagami M, Tomlinson LA, Mansfield KE, McDonald HI, Smeeth L, Nitsch D (2017). Prevalence, incidence, indication, and choice of antidepressants in patients with and without chronic kidney disease: a matched cohort study in UK Clinical Practice Research Datalink. Pharmacoepidemiol Drug Saf.

[8] De Hert M, Dekker JM, Wood D, Kahl KG, Holt RI, Möller HJ (2009). Cardiovascular disease and diabetes in people with severe mental illness position statement from the European Psychiatric Association (EPA), supported by the European Association for the Study of Diabetes (EASD) and the European Society of Cardiology (ESC). Eur Psychiatry.

[9] Pastoor D, Gobburu J (2014). Clinical pharmacology review of escitalopram for the treatment of depression. Expert Opin Drug Metab Toxicol.

[10] Boulenger JP, Huusom AK, Florea I, Baekdal T, Sarchiapone M (2006). A comparative study of the efficacy of long-term treatment with escitalopram and paroxetine in severely depressed patients. Curr Med Res Opin.

[11] Connolly KR, Thase ME (2016). Vortioxetine: a new treatment for major depressive disorder. Expert Opin Pharmacother.

[12] Tovilla-Zárate CA, Pérez-Mandujano A, Ramírez-González IR (2019). Vortioxetine versus sertraline in metabolic control, distress and depression in Mexican patients with type 2 diabetes. Ann Transl Med.

[13] Baldwin DS, Necking O, Schmidt SN, Ren H, Reines EH (2022). Efficacy and safety of vortioxetine in treatment of patients with major depressive disorder and common co-morbid physical illness. J Affect Disord.

[14] De Diego-Adeliño J, Crespo JM, Mora F, Neyra A, Iborra P, Gutiérrez-Rojas L, Salonia SF (2022). Vortioxetine in major depressive disorder: from mechanisms of action to clinical studies. An updated review. Expert Opin Drug Saf.

[15] Hamilton M (1960). A rating scale for depression. J Neurol Neurosurg Psychiatry.

[16] (2023). The R Project for Statistical Computing. https://www.R-project.org/.

[17] Santi NS, Biswal SB, Naik BN, Sahoo JP, Rath B (2023). An interim analysis of a randomized, open-label study of vilazodone, escitalopram, or vortioxetine for major depressive disorder. Cureus.

[18] Santi NS, Biswal SB, Naik BN, Sahoo JP, Rath B (2023). Quality of life and medication adherence in patients with major depressive disorder: an interim analysis of a randomized study. Cureus.

